# Biofabrication and spectral characterization of silver nanoparticles and their cytotoxic studies on human CD34 +ve stem cells

**DOI:** 10.1007/s13205-016-0532-5

**Published:** 2016-10-06

**Authors:** Venkata S. Kotakadi, Susmila Aparna Gaddam, Sucharitha K. Venkata, P. V. G. K. Sarma, D. V. R. Sai Gopal

**Affiliations:** 1 DST-PURSE Centre, Sri Venkateswara University, Tirupati, Andhra Pradesh India; 2 Department of Virology, Sri Venkateswara University, Tirupati, Andhra Pradesh India; 3 Department of Home Science, Sri Venkateswara University, Tirupati, Andhra Pradesh India; 4 Department of Biotechnology, SVIMS, Tirupati, Andhra Pradesh India

**Keywords:** Biosynthesis, Silver nanoparticles, Cytotoxic studies, CD34 +ve stem cells, Microcarrier culture

## Abstract

Biosynthesis of plant-mediated silver nanoparticles is gaining significant importance due to environmentally safe ‘green method’ and it is an efficient alternative method. In the present study, silver nanoparticles were synthesized by using root extract of *Glycyrrhiza glabra* an important medicinal plant. The AgNPs are characterized by spectral analysis; the surface plasmon resonance (SPR) peak of AgNPs showed maximum absorption at 445 nm. Fourier-transform infrared spectroscopy (FT-IR) data show that the O–H hydroxyl groups, carboxylic acids, ester and ether groups and C–O stretching of alcohols have been utilized in the formation of AgNPs. The X-ray powder diffraction (XRD) data reveal that the AgNPs are face-centered cubic (fcc) in structure. The size was determined by particle size analyzer and atomic force microscope (AFM); the results reveal that AgNPs were spherical in shape and the average grain size is determined as 41.5–46.5 nm. Transmission electron microscopy (TEM) micrographs obtained show that AgNPs were roughly spherical and well dispersed with the sizes ranging from 10 to 45 nm ± 5 nm. The biofabricated AgNPs are extremely stable due to its high negative zeta potential −34.1 mV which indicates that the nanoparticles are polydispered in nature. The cytotoxic studies of AgNPs on human CD34 +ve stem cells in microcarrier culture reveal excellent growth at different concentrations of biosynthesized AgNPs. This is the first report of microcarrier culture of CD34 +ve stem cells on biosynthesized AgNPs.

**Electronic supplementary material:**

The online version of this article (doi:10.1007/s13205-016-0532-5) contains supplementary material, which is available to authorized users.

## Introduction

In modern research, nanotechnology is the current area of materials science. In medical technology, green synthesis of nanomaterials is gaining importance. The application of silver nanoparticles has a wide range, which gives scope for further research in the field of fabrication of silver nanoparticles and their compounds of ionic silver (Willner et al. 2006; Lou et al. 2006). Some of the very important applications of silver nanomaterials include antimicrobial properties, surface-enhanced Raman scattering effect and also catalytic activity (Li et al. 2006; Chen et al. 2005). It is already known that silver has been used as an antimicrobial agent for centuries, in the present days the repetition of antibiotics increasing threat of antibiotic resistance, caused by the exploitation of antibiotics. So, Scientists once again focus on the application of silver nanoparticles (AgNPs) (Sambhy et al. 2006). Besides antimicrobial activity, silver nanoparticles have potential applications as intercalation material in batteries, as coatings in solar energy, in optical receptors, in bio-labeling, and as efficient biocatalysts in chemical reactions (Klaus-Joerger et al. 2001; Schultz et al. 2000; Hayat 1989). As the result, the biologists developed interest in the field of nanotechnology and focused on new dimensions of research in nanoparticles synthesis and their applications are emerging at rapid pace as therapeutic tools (Simkiss and Wilbur 1989; Mann 1996; Kotakadi et al. 2013, 2014, 2015a, b; Gaddam et al. 2014). Thus, the biosynthesized AgNPs have a wide range of significant applications in bimolecular diagnostics, competent antimicrobial, therapeutics, catalysis and also in microelectronic devices (Litvin and Minaev 2013; Schultz et al. 2000; Rai et al. 2009). Earlier reports on the synthesis of AgNPs by using synthetic humic acids reveals that the nanoparticles are very stable and can be useful in different biomedical applications (Litvin and Minaev 2013; Litvin et al. 2012). Since the last decade, regenerative medicine is gaining a lot of attention from use of stem cells in cell and tissue replacement therapeutics (Delcroix et al. 2009; Huang et al. 2009; Heymer et al. 2008). There are several challenges in stem cell therapy; to overcome these difficulties, the bionanoparticles were widely used due to their biocompatibility nature. Thus, these bionanoparticles are highly essential in biomedicine and also for evaluating their potential toxicity (Park et al. 2007; Huang et al. 2008). The nanoparticles are very minute in nature, because of their tiny nature more than 10 millions of them are ingested by each and every human being daily and they easily circulate through the body (Nel et al. 2006). As of now very little data are available on cytotoxic studies of biosynthesized silver nanoparticles on stem cells. The cytotoxicity depends upon concentration of nanoparticles (Braydich-Stolle et al. 2005). Thus, the present work was carried out to study the cytotoxicity of green synthesized silver nanoparticles at different concentrations on human CD34 +ve stem cells.

## Materials and methods

### Synthesis of silver nanoparticles

The *Glycyrrhiza glabra* root powder (Fig. S. see Supplementary data) was collected from Sri Venkateswara Ayurveda College of Pharmacy, Srinivasa mangapuram, Tirupati, Andhra Pradesh, India. The root extract was prepared, by taking 1 g of finely powdered roots with 100 ml of sterile Milli-Q water in a sterile 250 ml conical flask and mixed thoroughly; the mixture was heated at 70 °C for 10 min. After 10 min the sample was filtered through sterile muslin cloth followed by Whatman no.1 filter paper. The filtrate of the extract was used to carry out the synthesis of silver nanoparticles. To 1 ml of root extract filtrate, 4 ml of sterile Milli-Q water and 10 ml of 0.025 (M) AgNO_3_ were added and left at room temperature and the reaction was observed. The colorless root extract was reduced by AgNO_3_ and the color of the solution changed to yellow to dark brown (see Fig. S.1. in Supplementary data). The color change indicated the reduction of silver ions into AgNPs. The earlier studies also reveal that AgNPs exhibit a dark brown color in aqueous solution due to surface plasmon resonance (SPR). In the present study, the AgNPs were synthesized by *Glycyrrhiza glabra* root extract without any toxic chemicals. Thus, this method is known as eco-friendly, environmental safe “Green method”.

### Spectral characterization

The biosynthesized AgNPs with the root extract of *Glycyrrhiza glabra* were analyzed using Nanodrop (UV–Visible spectrophotometer, Thermo Scientific). The optical absorbance of the AgNPs was recorded at wave length range 200–700 nm by periodically sampling 1–3 µl of the sample, and the reaction of the sample was carried out at room temperature on the Nanodrop spectrophotometer at 1 nm resolution. FT-IR analysis was carried out by Alpha T model, FT-IR spectrophotometer, Bruker Company. The synthesized AgNPs were carefully prepared by centrifuging at 9000 rpm for 20 min and the pellet was washed thoroughly with sterile Milli-Q distilled water thrice to remove the unbound plant extract residues. The isolated AgNPs were used for IR analysis. The particle size analysis and Zeta potential measurement experiments were carried out by Horiba SZ-100 nanoparticle analyzer. The particle size was performed by dynamic light scattering (DLS) of nanoparticles present in the solution, and the charge on the surface of the AgNPs was also measured by SZ-100. Further, the size and morphology of the synthesized AgNPs were also done by atomic force microscope (AFM-Solver Next, NT-MDT, Russia). The AFM analysis was carried out by coating a thin film of AgNPs on a sterile clean glass cover slip and it was air-dried prior to the analysis. The shape and size of the AgNPs were also determined by using transmission electron microscopy studies (TEM-FEI Tecnai F12, Philips Electron Optics, Holland) operated at 100 kV. The sizes of the synthesized silver nanoparticles were determined by using SIS imaging software (Munster, Germany).

### Cell culture

The institutional ethical committee has cleared in vitro differentiation studies on human CD34^+^ stem cells (Sarma and Subramanyam 2008). These cells were isolated and sub-cultured in tissue culture flask with DMEM containing 10 % FBS and cultured at 37 °C with 5 % CO_2_ atmosphere and 95 % humidity for 4 days. The cells were allowed to grow till they reached confluent state. The grown cell culture was treated with 0.25 % trypsin to digest the cell adherent proteins so as to obtain the isolated cells (Ian Freshney 2005). The trypsin activity was arrested by the addition of excess serum (FBS). Then the cells collected were allowed to settle by centrifuging at 1200*g* for 3 min at room temperature. The pelleted cells were washed with plain DMEM and the cell count was performed using a hemocytometer. Then the cells were seeded into a fresh culture flask with serum-free plain DMEM and this fresh serum free cell culture was maintained at 37 °C with 5 % CO_2_ atmosphere and 95 % humidity for 7 days. Before going to co-culturing with *Glycyrrhiza glabra* root extract synthesized bionanoparticle. The cells were passaged regularly when they reached >80 % confluency. The purity and cell viability was assessed by May–Grünwald–Giemsa stain method and 0.4 % trypan blue, respectively.

### Characterization of cultured cells

The CD34^+^ cells were characterized by performing ImmunoCytoChemistry (ICC) study. The cells were smeared over a clear glass slide and fixed with 4 % paraformaldehyde at 37 °C for 1 h. The slides were washed with 1XPBS till no pungent smell was observed. Then the slides were treated with the blocking buffer to mask the non-specific sites. Again the slides were washed with PBS to remove excess blocking buffer. Then the slides were treated with 3 % H_2_O_2_ to saturate the endogenous peroxidase effect. The slides were washed with PBS for two times and primary antibody against CD34 antigen raised in mouse (Qbend CLONE 10 obtained from Dako) was overlaid on the slide and it was incubated for 1 h at room temperature, followed by addition of secondary antibody, which was conjugated with streptavidine-HRPO with an incubation of 1 h at room temperature. Then the developer was added to the slide which was prepared with DAB and H_2_O_2_. Cells were observed under microscope.

### In vitro assay for cytotoxic activity of AgNPs

MTT assay: (MTT = “3-(4,5-Dimethylthiazol-2-yl)-2,5- diphenyl tetrazolium bromide”).

Cell viability was quantified by performing MTT assay as described by Ian Freshney (2005). In brief, cells were seeded in micro-titer plate at a seeding density of 10 cells, 20 cells, 40 cells, 80 cells and 160 cells per well in the respective rows followed by 2 days’ incubation at 37 °C with 5 % CO_2_ and 95 % humidity. The supernatant was aspirated from the wells, and fresh aliquots of growth medium having *Glycyrrhiza glabra* root extract synthesized bionanoparticles at a concentration of 1, 2, 4, 8, 16 μl were added from the sub-stock of 1:100 diluted main stock. After overnight incubation the supernatant was aspirated, and cell monolayers were washed with PBS. Subsequently, 50 µg (i.e., 10 µl) of MTT solution from 5 mg/mL stock was added in each well, incubated for 16 h and the supernatant was removed. To each well 200 µl DMSO + Sorensen’s glycine buffer (0.1 M glycine, 0.1 M NaCl adjusted to pH 10.5 with 1 M NaOH) were added to solubulize the crystals formed by MTT in each well of the microtitre plate the samples were read at 570 nm and the absorbance was recorded (Tables 1, Supporting Information).

### Scanning electron microscopy of stem cell aggregates

Scanning electron microscopy (SEM) was performed by Oxford Inca Penta FeTX3 EDS instrument attached to Carl Zeiss EVO MA 15 Scanning Electron Microscope (200 kV) machine with a line resolution 2.32 (in Å) of the SEM images of CD34 +ve cells

### Results and discussion

The bioactive constituents from medicinal plants are used as new drugs directly or indirectly. *Glycyrrhiza glabra* **(**Licorice), the most important approved herb after ginseng in China, is used for ailments related to spleen, liver and kidney, and the Japanese use the herb as an antiviral agent. The underground unpeeled or peeled stems or roots are used for the treatment. Licorice is widely used in polyherbal formulations and most of them have been evaluated during clinical studies (Dhanaukar et al. 2000). Liquorice has Glycyrrhizin as the major water-soluble constituent responsible for its sweet taste. Glycyrrhizin is a triterpenoid saponin that is present within range 2–14 %. Phytochemicals present in liquorice are flavanones, chalcones, isoflavones, glycyrrhizoflavonone, licoisoflavanone (isoflavanones), glycocoumnarins, lipocoumarins, glycyrin, and isoglycryol. *G. glabra* is used in folk medicine as a laxative, contraceptive, galactagogue, anti-asthmatic drug and antiviral agent. Due to the vast range of biological effects like anti-inflammatory, anti-allergic, antioxidant, antiviral of the phytochemicals present in extracts has been of immense importance in phytotherapeutics.

### UV–Visible analysis

**Fig. 1:**
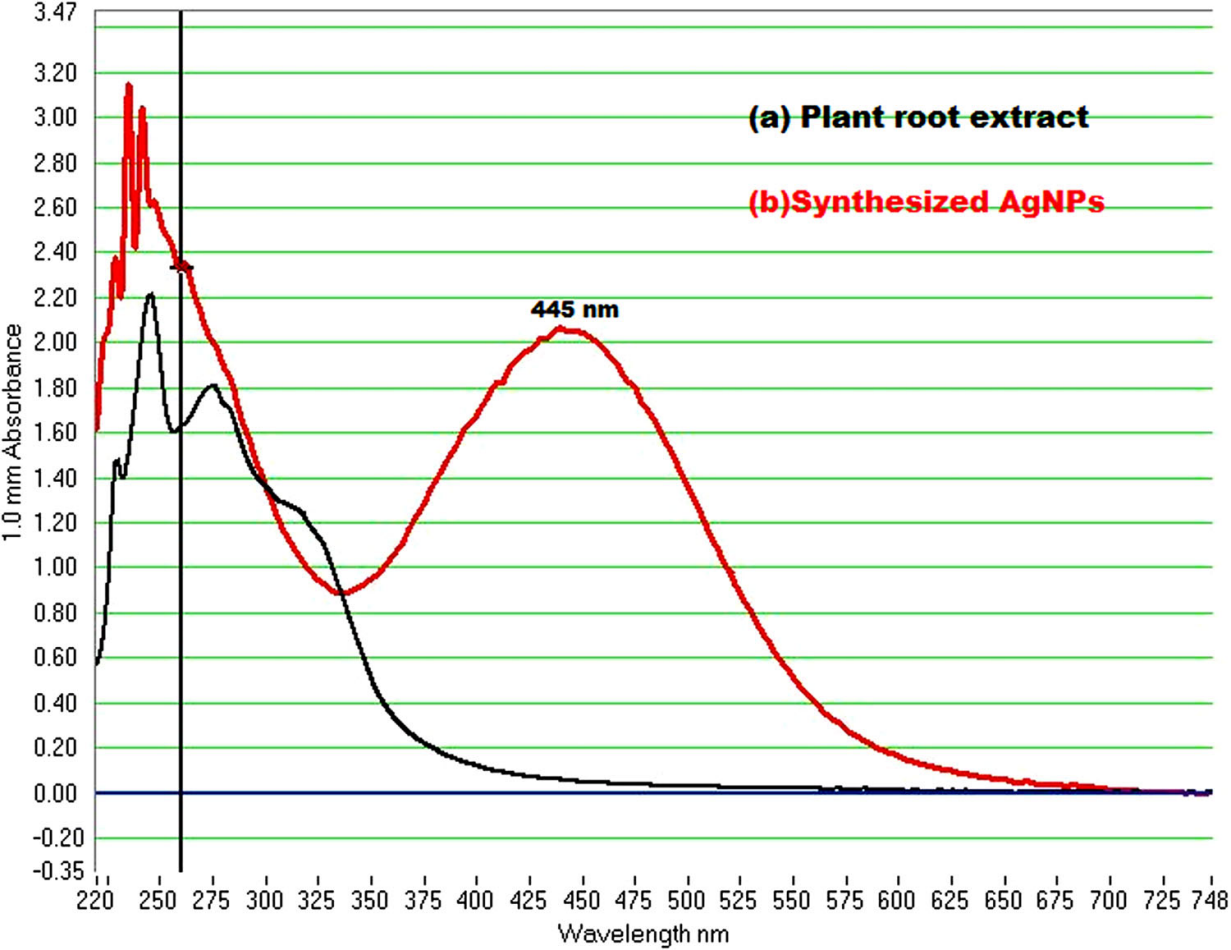
UV-VIS absorbtion spectra of AgNps synthesized from *G. glabra* leaves extract with 2 × 10^−3^ M silver nitrate

The green synthesized AgNPs were measured using UV–Visible Nanodrop 8000 spectrophotometer from 220 nm to 750 nm wavelength range, and the surface plasmon resonance (SPR) spectra of AgNPs were obtained at 445 nm (Fig. 1). The SPR was due to the presence of free electrons present on AgNPs, and it also plays a vital role on the size of the synthesized nanoparticles (Mock et al. 2002) Previous reports reveal that the nanoparticles having SPR range between 390 and 420 nm have been reported to have small nanoparticles, i.e., AgNPs around 25 nm -50 nm in size (Panacek et al. 2006) and also particles having SPR around 450 nm and above have variable size of AgNPs from 2 to 100 nm and above (Vijayaraghavan et al. 2012). The AgNPs are spherical in shape, which are reported have the SPR around 410–450 nm (Sivalingam et al. 2012; Mock et al. 2002; Zaheer and Rafiuddin 2012), moreover longer the wavelength, the size of nanoparticles is increased, shorter the wavelength, smaller the size of the AgNPs (Kannan et al. 2011). The SPR of biosynthesized AgNPs by root extract is around 445 nm, so with the result we can assume that the synthesized nanoparticles are spherical in shape and may be in the range of 25 nm ± 10 to 100 nm ± 20 nm in range. Our other studies will confirm the results regarding the size and shape of synthesized nanoparticles.

### FT-IR analysis

**Fig. 2:**
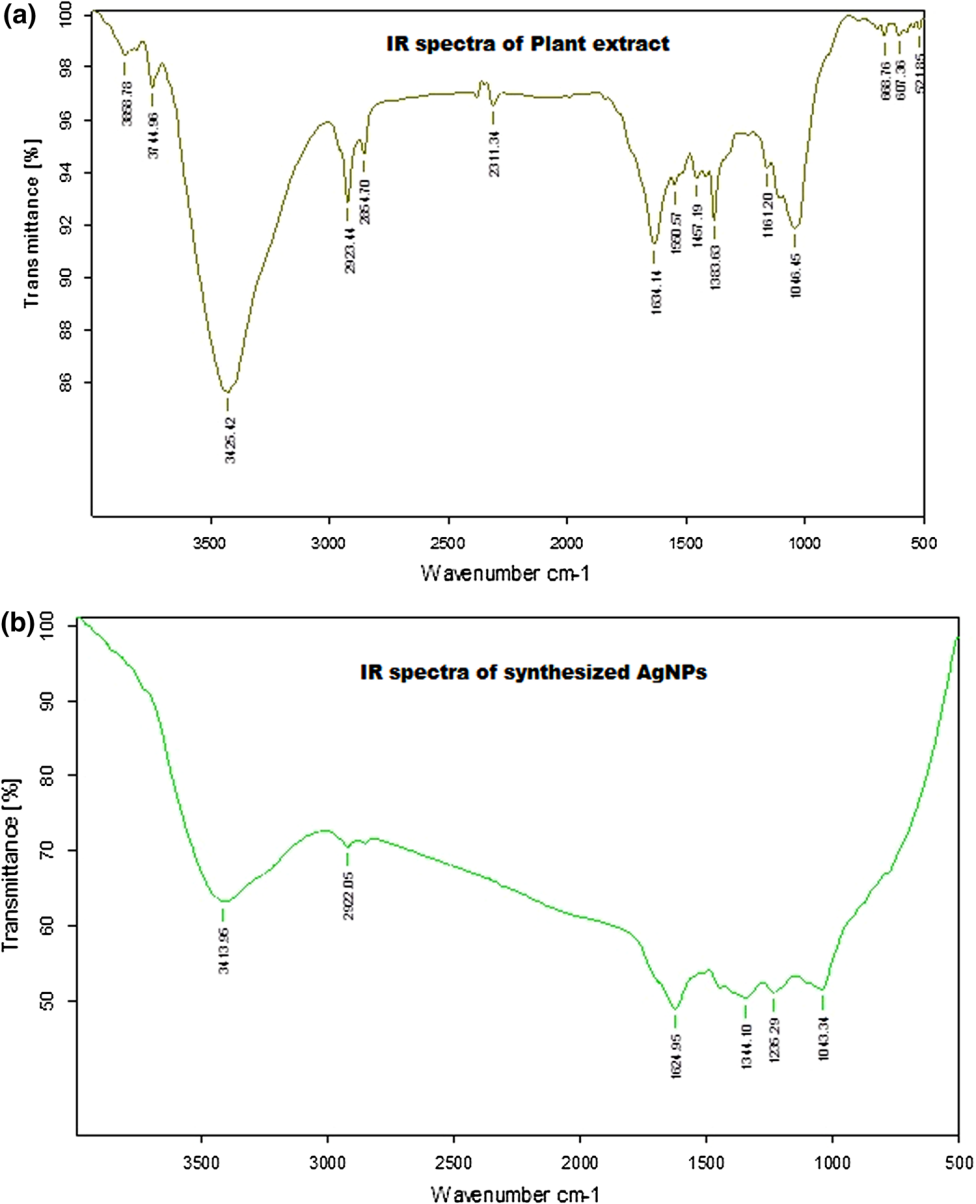
**a** IR spectrum of the root extract of *G. glabra*, **b** IR spectrum of the synthesized AgNps

In green synthesis the plant root extract plays dual role in synthesis of AgNPs both as capping agent and as bioreductant in the reaction. After the synthesis of AgNPs they are purified by centrifuging at 9000 rpm for 20 min and the pellet of AgNPs was collected. The centrifugation step was repeated thrice by washing the pellet with sterile distilled water to remove the any unbound residues of the plant root extract of *Glycyrrhiza glabra*. The purified AgNPs are now used for IR analysis. The IR results reveals that the spectra of root extract and the AgNPs have more similarities, confirming that the plant root extract components like flavonoids and poly phenols are involved in the bioreduction and formation of AgNPs. The IR spectrum reveals that there are some marginal shifts in the peak positions of AgNPs, which is due bioreduction. Further the FT-IR data was analyzed to distinguish the possible bio-reducing biomolecules present in the plant root extract. The FT-IR spectra of root extract Fig. 2a reveals strong spectral peaks at 3744, 3425, 2923, 2854 and 2311 cm^−1^, 1634, 1550 and 1457 cm^−1^, 1383, 1161 and 1046 cm^−1^ of different functional peaks. Whereas the AgNPs, Fig. 2b showed strong characteristic bands of different functional groups at 3413, 2922, 1624, 1344 and 1043 cm^−1^.

The results are compared among the strong and broad peaks of root extract and biosynthesized AgNPs are as follows i.e. 3425 and 3413 cm^−1^ which corresponds to –NH stretching in amide II and also the hydrogen-bonded hydroxyl (OH), were observed in both the extract and AgNPs. The peaks at 2923 and 2922 cm^−1^ corresponds to asymmetric stretching of C–H group and the other peaks corresponding to 1634 and 1624 cm^−1^ were identified as the characteristic peaks for the C–H, C–C, C–O stretching and the amide I group due to the carbonyl vibrational stretches in amide group linkages in proteins. The peaks at 1383 and 1344 cm^−1^ bands corresponds to C–N stretching of aromatic amines and finally the intense peaks at 1046 and 1043 cm^−1^ are the bands corresponding to characteristic peaks of C–OH stretching of secondary alcohols, carboxylic acids, ester and ether groups.(Shameli et al. 2012; Kora et al. 2012). It is already known that the noble metal nanoparticles have useful functional groups on the surface to form a protective layer and gives stability to the nanoparticles. The functional groups such as amino (–NH), carboxylic acid (COOH), mercapto (SH) and cyano (CN) and other groups have been proved to have high affinity towards metal nanoparticles (Teranishi et al. 1998; Corbierre et al. 2001; Mandal et al. 2002; Shan et al. 2003). In the present study we identified that different functional groups such as –NH, OH, C–H, C–C, C–O have been actively participated in the synthesis of silver nanoparticles using the root extract of *Glycyrrhiza glabra*. *Glycyrrhiza glabra* has Glycyrrhizin as the major water-soluble constituent. Glycyrrhizin is a triterpenoid saponin that is present within range of 2–14 %. So the water soluble Glycyrrhizin and other bioactive components may have actively participated in synthesis of AgNPs.

### XRD analysis

**Fig. 3:**
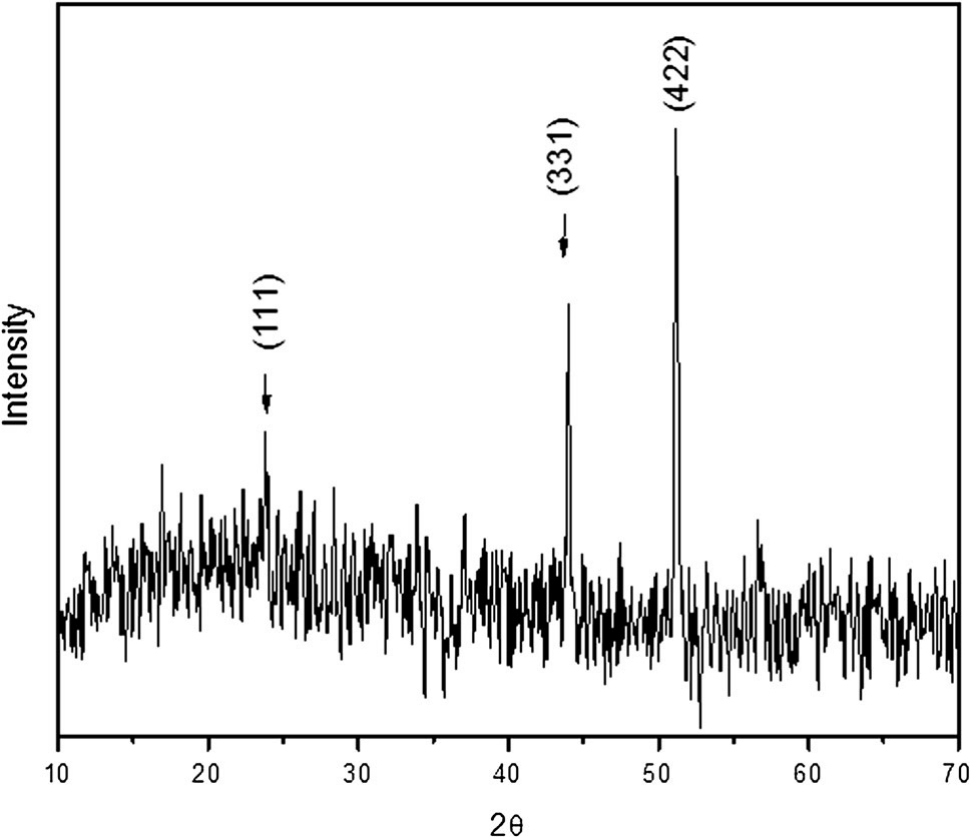
XRD spectral data of synthesized silver nanoparticles

X-ray diffraction studies of biosynthesized AgNPs was carried out to understand the crystalline nature of the particles. The XRD pattern of synthesized AgNPs in shown in Fig. 3, it reveals three distinct reflections in the diffractogram at at 23.83° (111), 44.14° (311), and 51.15° (422), respectively, which indicates that the particles are crystalline in nature with face central cubic structure. And it is also concluded that there are no additional reflections other than Ag lattice, thus indicates that the AgNPs are very stable and are not affected by any other molecules present in the root extract (Philip 2011).

### Particle size analysis

**Fig. 4:**
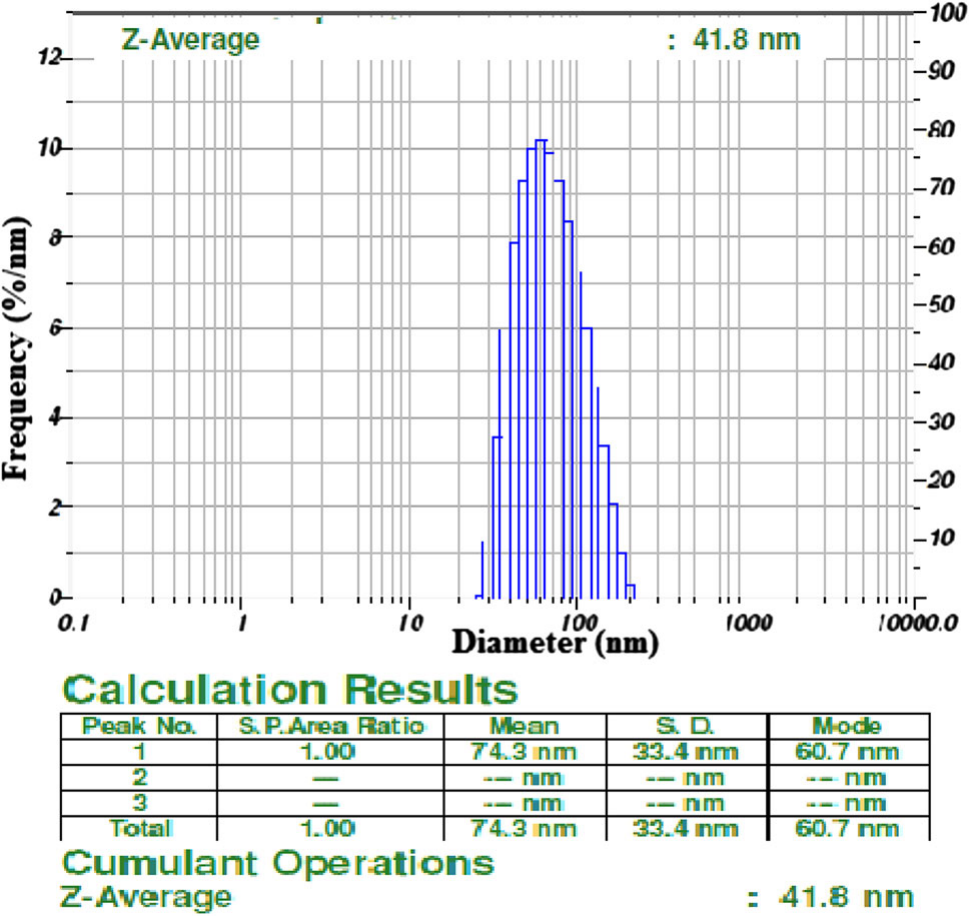
Particle size distribution curve for AgNPs

The AgNPs particle size was determined by Nanoparticle size analyzer by intensity and laser diffraction method, the results reveals that the biosynthesized AgNPs are of varied size in nature ranging from 33.4 to 74.3 nm in diameter. The average size of the AgNPs was found to be 41.8 nm (Fig. 4) and it reveals that the particles are poly dispersed in mixture solution.

### Zeta potential measurement

**Fig. 5:**
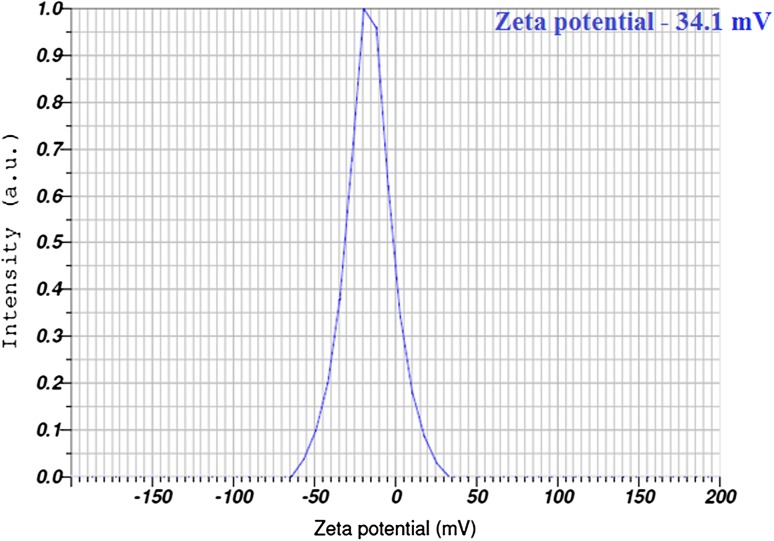
Zeta potential of synthesized AgNPs

The biosynthesized AgNPs found to have negative zeta potential −34.1 mV (Fig. 5), which indicates the repulsion among the green synthesized silver nanoparticles and increase the stability of the formulation. It is evident that the AgNPs are poly dispersed in nature due its high negative zeta potential thus the electrostatic repulsive force between them results in the prevention of agglomeration of the nanoparticles and also very much helpful for long term stability in the solution (Suresh et al. 2011).

### AFM analysis of AgNPs

**Fig. 6:**
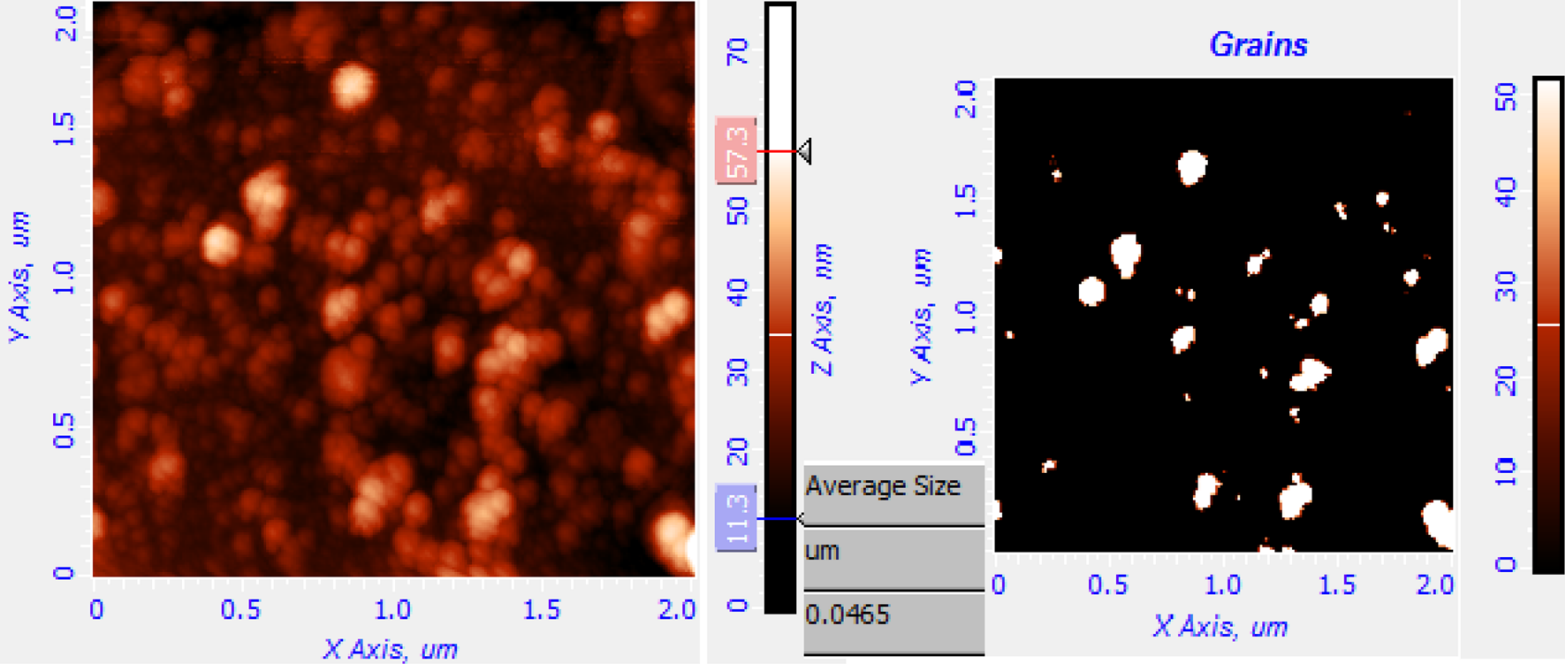
a 2D image of synthesized AgNps, **b** grains detected

Atomic force microscopy is an advanced spectroscopic method used to analyze the biosynthesized AgNPs to find out the morphology and image topology of the nanoparticles. The biosynthesized AgNPs were coated as a thin film on a piece of thick aluminum foil and analysis was carried out using AFM by tapping mode. The results reveal that the particles topology and morphology appears to be spherical in shape (Fig. 6). The grain analysis of the AFM image was carried out using Nova-Px 3.2.0.rev soft ware provided by NT-MDT. The grain size of the silver nanoparticles was found to be varied in size that is 36–46.5 nm and above, and the average grain size was found to be 46.5 nm.

### TEM analysis

**Fig. 7:**
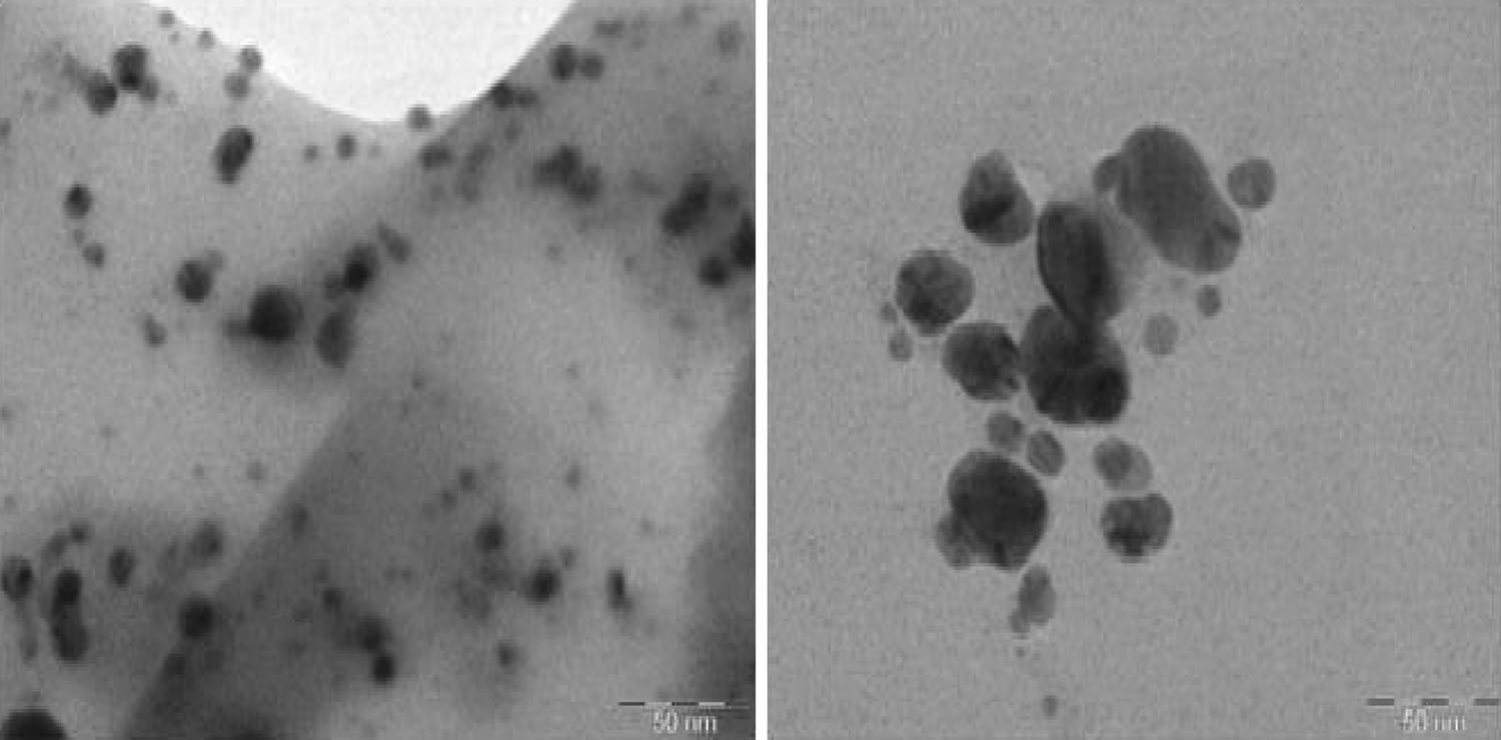
TEM analysis of synthesized AgNPs

Transmission electron microscopy (TEM) analysis of the AgNPs shows that the synthesized particles were roughly spherical in shape and were well dispersed in nature with varied size ranging from 10 to 45 nm ± 5 nm (Fig. 7). Finally, it is concluded that the size and the shape of the AgNPs were fairly uniform and similar to the earlier experiments carried out in our study. At the same time TEM studies were consistent with earlier reports.

### In vitro assay for cytotoxic activity of AgNPs

**Fig. 8:**
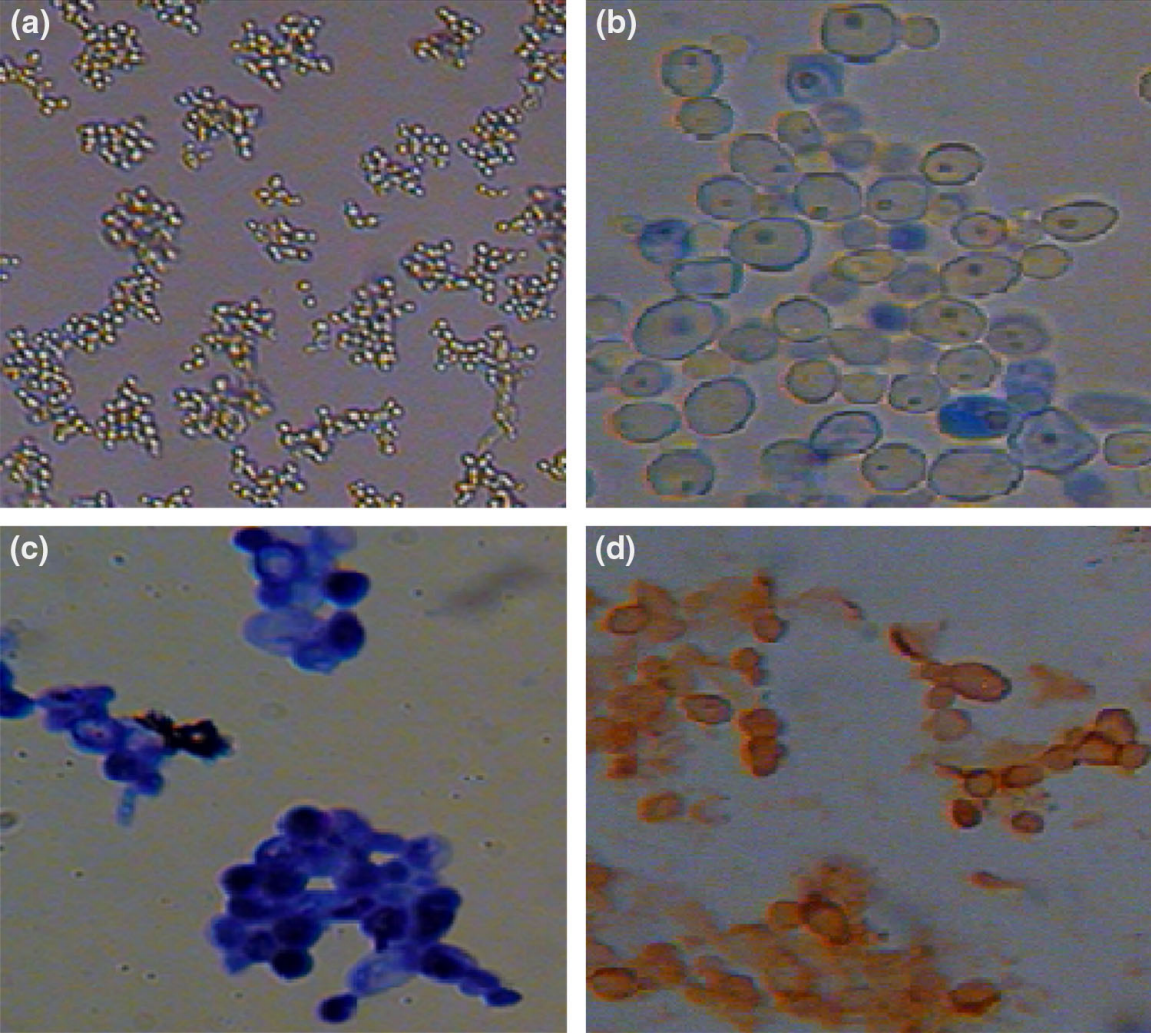
**a** Cultured human CD34^+^ cells; **b** Giemsa-stained cells; **c** Trypan blue tested cells. **d** ICC stained cells

The cultured cells obtained as monolayer showed that nucleated structures were observed after staining with May–Grünwald–Giemsa stain. As observed from the trypan blue dye exclusion method, cell viability showed >85 %. From the ICC study, the presence of CD34+ antigen marker on cells was confirmed (Fig. 8a–d). From MTT assay the cytotoxicity of nanoparticles was assessed where it shows the 107 % proliferation rate (PR %) which was determined by considering the positive control as 100 %.

- PR % = [(Cells cultured with AgNPs RPR %)/(positive control RPR %)] × 100
- RPR % = RPR × 100
- The relative proliferation rate (RPR) was found to be 70.4230 from the formula RPR = (*A* − *A*_*N*_)/*A*_*N*_, where ‘*A*’ represents the absorbance of cell culture grown with sample 1; ‘*A*_*N*_’ was the absorbance of negative control (DMEM + nanoparticle stock). **(**see supporting information and Table 2, Table 3 and Figs. S2, S3 supporting information)

### Scanning electron microscopy of stem cell aggregates

**Fig. 9:**
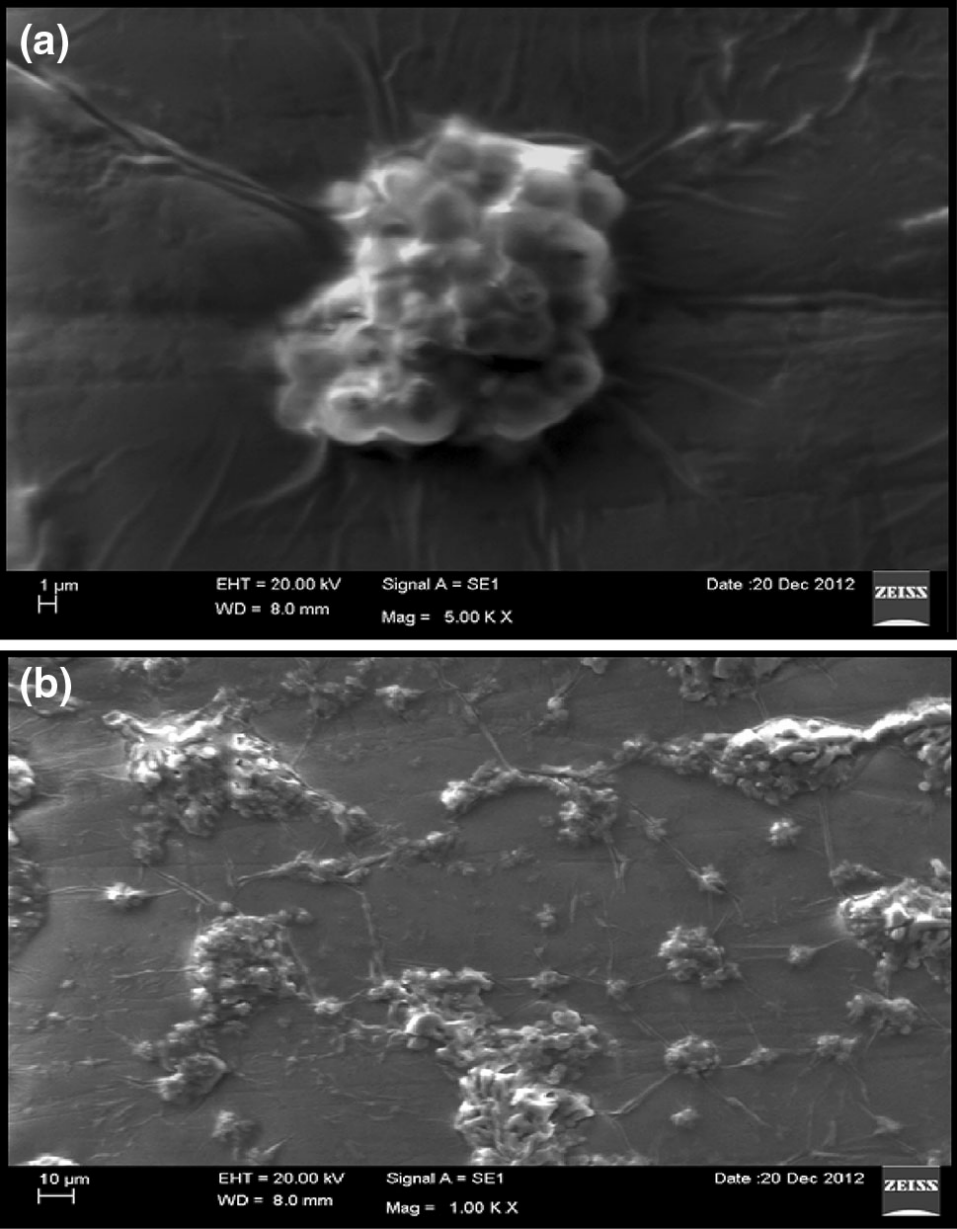
**a** SEM image cells aggregate over Ag nanoparticles. **b** SEM image stem cell culture with Ag nanoparticles

The morphology and shape of the stem cells, after treatment with different concentrations of biosynthesized AgNPs were analyzed by scanning electron microscopy (SEM), (Oxford Inca Penta FeTX3 EDS instrument attached to Carl Zeiss EVO MA 15 Scanning Electron Microscope (200 kV) machine with a line resolution 2.32 (in Å)). The SEM images of CD34 +ve cells along with AgNPs reveal that the differentiation and multiplication of cell was very good and there was no cytotoxicity (Fig  9a, b).

**Fig. 10:**
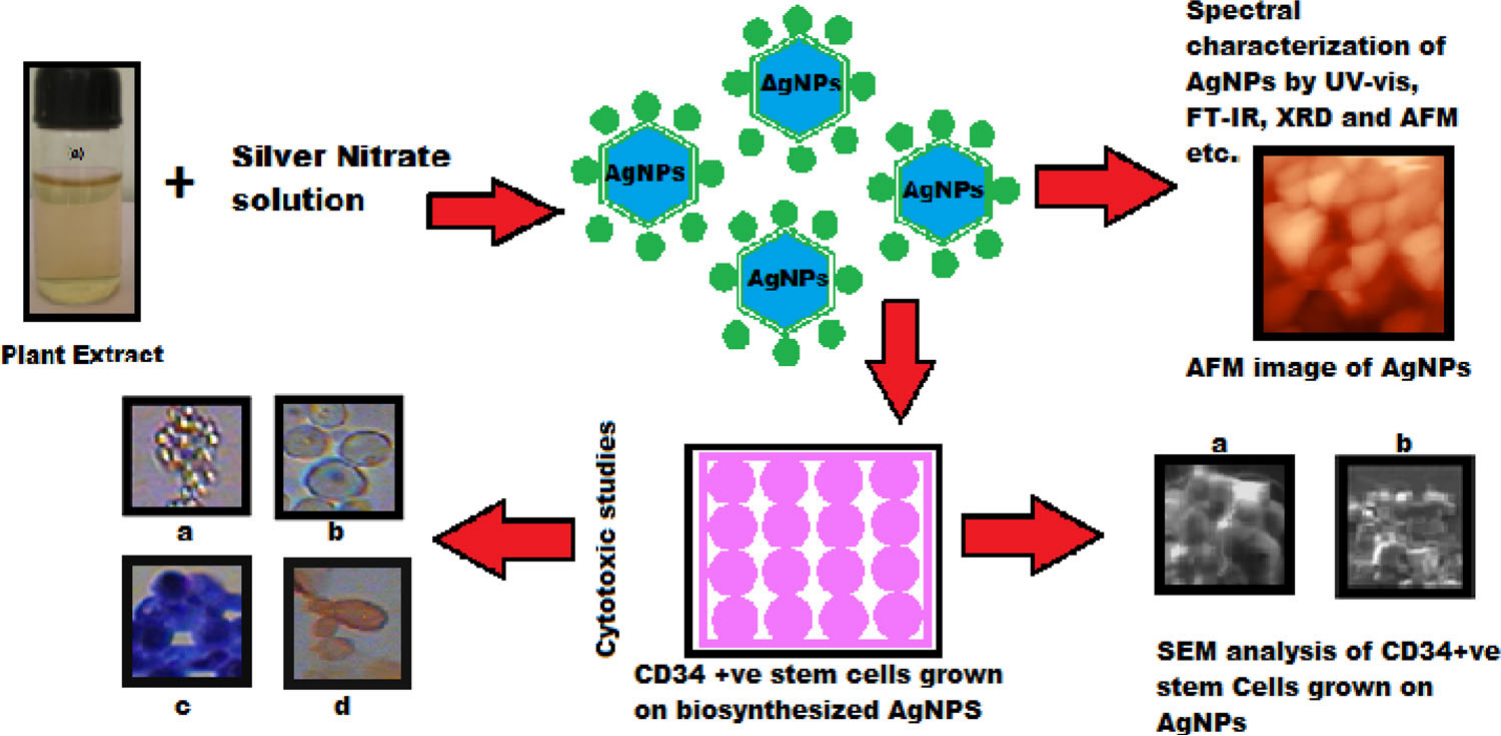
Schematic illustration of the green synthesis of silver nanoparticles (AgNPs) using aqueous extract of the *Glycyrrhiza glabra* plant roots

*G. glabra* root extract is found to be exceptionally suitable for rapid synthesis of silver nanoparticles by toxic free green method within 5 to 10 min. Further the AgNPs are characterized by different spectroscopic methods like UV–Vis, FT-IR, XRD, particle size and Zeta potential, TEM and AFM analysis and also SEM analysis of the stem cells of the present study. From the above results it is clearly understood that the AgNPs are highly stable and polydispersed in nature with an average size of 41.1 ± 5 to 45.5 ± 1 nm. The synthesized AgNPs are less than 50 nm in size due to their small size the AgNPs can be useful in various biomedical applications. The nanoparticles have high surface-to-volume ratio due to their physical and chemical properties; thus they are easily tailorable, multi-functional and have high solubility (Azzazy and Mansour 2009). The cytotoxic effects of different types of metallic nanoparticles on stem cells have been studied by earlier by scientists and researchers. When cadmium oxide nanoparticles were added to stem cells germ lines at the concentration of 1 μg/ml, the stem cell growth was inhibited by cell shrinkage, thus the cells become irregular in shape and mitochondrial dysfunction occurs. But when the concentration of nanoparticles increased to 5 μg/ml, the stem cells showed drastic changes, cells become necrotic and they are detached from the cultured dishes (Braydich-Stolle et al. 2005). Whereas when human bone marrow mesenchymal stem cells treated with mesoporous silica nanoparticles conjugated with fluorescein isothiocyanate, do not show any toxicity, the stem cells are viable and highly proliferate (Chung et al. 2007). Similarly other studies with super paramagnetic iron oxide nanoparticles reveal that the stem cells have a high percentage of survival rate of 91–99 %, indicating that the iron oxide nanoparticles do not affect stem cell viability (Delcroix et al. 2009; Heymer et al. 2008; Jing et al. 2008; Wang et al. 2009). The cytotoxicity of nanoparticles on stem cells depends on the type of nanoparticles. Whereas in the present study, the biosynthsised AgNPs by green route using *G. glabra* root extract do not show any cytotoxic effect on human CD34 +ve stem cells. The human CD34 +ve stem cells are proliferated very efficiently. So we hereby report for the first time the synthesis of silver bionanoparticles which do not show any cytotoxicity on stem cells. The whole study is illustrated in a schematic diagram which is Fig. 10.

## Conclusion

In the present study, the biosynthesized silver nanoparticles are smaller than 50 nm which can play a very important role in biomedical applications. So the human CD34 +ve stem cells grown on silver nanoparticles can be useful to treat chronic diseases and are much useful in regenerative medicine for repairing the tired and failing organ systems. Hence, the biosynthesised nanoparticles by using *G. glabra* root extract can play a very important role in regenerative medicine and clinical therapies like wound healing and in vitro growing of organs and tissues for various chronic diseases.

## Electronic supplementary material

Below is the link to the electronic supplementary material.

Supplementary material 1 (DOCX 236 kb)
