# Supplementary material for: Biofabrication and spectral characterization of silver nanoparticles and their cytotoxic studies on human CD34 +ve stem cells
Source: 3 Biotech. 2016 Oct 6;6(2):216. doi: 10.1007/s13205-016-0532-5 (PMC5053953; doi:10.1007/s13205-016-0532-5)
Supplement: Supplementary file 1 — Supplementary material 1 (DOCX 236 kb) [file 13205_2016_532_MOESM1_ESM.docx]

**Supplementary data**

**
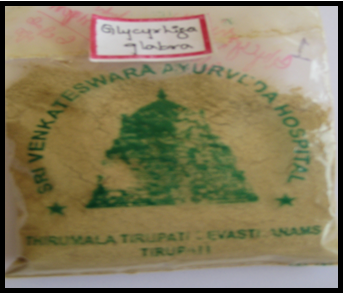
**

**Fig. S.** *Glycyrrhiza glabra* root powder

**
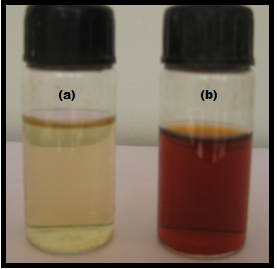
**

**Fig. S1** in Supporting Information

**a).** *Glycyrrhiza glabra* root extract

**b),** AgNPs synthesized by *Glycyrrhiza glabra* root extract

| **Samples** | **OD @ 570nm** |
| --- | --- |
| **Cells cultured with AgNps (Sample1)** | **1.857** |
| **positive control(DMEM+ Stem cells)** | **1.732** |
| **negative control(DMEM+ Nano particle)** | **0.026** |
| **negative control(Only DMEM)** | **0.019** |

**Tables.1,** Supporting Information

**Fig. S2** in Supporting Information

| **Samples** | **OD @ 570nm** | **Net OD*** | **RPR %** | **PR%** |
| --- | --- | --- | --- | --- |
| **Cells cultured with AgNPs(sample1)** | **1.857** | 1.831 | 7042.308 | **107.33** |
| **positive control(DMEM+ Stem cells)** | **1.732** | 1.706 | 6561.5384 | **100** |
| **negative control(DMEM+ Nano particle)** | **0.026** | 0 | 0 | **0** |
| **negative control(Only DMEM)** | **0.019** | -0.007 | -26.9230 | **-0.4103** |

*****Net Absorbance OD = (Absorbance of Cells) – (Negative Control Absorbance (DMEM+ Nano particle)

**Tables.2,** Supporting Information

| **Samples** | **PR%** |  |
| --- | --- | --- |
| **Cells cultured with AgNPs (sample1)** | **107.33** |  |
| **positive control(DMEM+ Stem cells)** | **100** |  |
| **negative control(DMEM+ Nano particle)** | **0** |  |
| **negative control(Only DMEM)** | **-0.4103** |  |

**Tables.3**, Supporting Information

**Fig. S3** in Supporting Information
